# Improving the Molecular Diagnosis of Malaria: Droplet Digital PCR-Based Method Using Saliva as a DNA Source

**DOI:** 10.3389/fmicb.2022.882530

**Published:** 2022-05-13

**Authors:** Gabriel Luíz Costa, Denise Anete Madureira Alvarenga, Anna Caroline Campos Aguiar, Jaime Louzada, Dhélio Batista Pereira, Tatiana Flávia de Oliveira, Antônio Augusto Fonseca Júnior, Luzia Helena Carvalho, Cristiana Ferreira Alves de Brito, Taís Nóbrega de Sousa

**Affiliations:** ^1^Molecular Biology and Malaria Immunology Research Group, Instituto René Rachou, Fundação Oswaldo Cruz (FIOCRUZ), Belo Horizonte, Brazil; ^2^Department of Bioscience, Federal University of São Paulo, Santos, Brazil; ^3^Health Sciences Center, Federal University of Roraima, Boa Vista, Brazil; ^4^Center for Research in Tropical Medicine, Porto Velho, Brazil; ^5^Laboratório Federal de Defesa Agropecuária de Minas Gerais, Pedro Leopoldo, Brazil

**Keywords:** malaria, molecular diagnosis, droplet digital PCR, saliva, *Plasmodium falciparum*, *Plasmodium vivax*

## Abstract

Malaria is an acute febrile disease caused by a protozoan of the genus *Plasmodium*. Light microscopy (LM) is the gold standard for the diagnosis of malaria. Despite this method being rapid and inexpensive, it has a low limit of detection, which hampers the identification of low parasitemia infections. By using multicopy targets and highly sensitive molecular techniques, it is possible to change this scenario. In this study, we evaluated the performance of droplet digital PCR (ddPCR) to detect *Plasmodium* DNA obtained from saliva samples (whole saliva and buccal swab) of 157 individuals exposed to malaria transmission from the Brazilian Amazon region. We used the highly sensitive ddPCR method with non-ribosomal multicopy targets for *Plasmodium vivax* (Pvr47) and *Plasmodium falciparum* (Pfr364). There was good concordance between the quantitative real-time PCR (qPCR) results from the saliva and blood, except for mixed-species infections. The sensitivity of qPCR was 93% for blood, 77% for saliva, and 47% for swabs. Parasite DNA was not detected in saliva samples in low-density infections compared with the detection in blood samples. ddPCR showed increased sensitivity for detecting *Plasmodium* in the blood and swabs (99% in blood, 73% in saliva, and 59% in swabs). Notably, ddPCR detected more mixed infections in the blood (15%), saliva (9%), and swabs (18%) than qPCR. Our data showed that the differences between ddPCR and qPCR were the result of a higher number of *P. falciparum* infections detected by ddPCR. Overall, there was a moderate correlation between parasite densities estimated by the different methods in the blood. Our findings highlight the possibility of using non-invasive sample collection methods for malaria diagnosis by targeting multicopy sequences combined with highly sensitive molecular methods.

## Introduction

Malaria is an acute febrile parasitic disease with major lethality. In 2020, there were an estimated 241 million malaria cases worldwide, representing an additional 14 million cases compared to those in 2019 (World Health Organization, 2021). These estimates include the impact of the COVID-19 pandemic on malaria prevention and treatment. However, in South America, the number of cases decreased from 894,000 in 2019 to 653,000 in 2020. In Brazil, the incidence decreased by approximately 10.5% during the same period (totaling approximately 141,000 cases in 2020, with 84% caused by *Plasmodium vivax* infection), even though almost 77% of all the cases reported in the Americas were from Brazil, along with Venezuela and Colombia (World Health Organization, 2020; BRASIL Ministério da Saúde, 2021).

The accurate diagnosis of malaria is fundamental for the adequate treatment of patients, prevention of mortality, and disease control. The gold standard for the diagnosis of malaria is parasite identification in Giemsa-stained thick blood smears via light microscopy (LM); however, this method has a moderate detection limit of ~50–100 parasites/μL under field conditions and could lead to a misdiagnosis of mixed infections (Kilian et al., 2000; Zimmerman and Howes, 2015). A cornerstone of malaria diagnosis and its elimination is the identification of submicroscopic infections of the two major species, *P. falciparum* and *P. vivax*, which act as reservoirs of the disease and are only detected with highly sensitive methods (Okell et al., 2012; Lindblade et al., 2013; Wampfler et al., 2013; Bousema et al., 2014; Vallejo et al., 2016). Molecular diagnostic methods are the most promising tools for detecting submicroscopic *Plasmodium* infections and for distinguishing mixed infections (Costa et al., 2014; Britton et al., 2016). To accomplish this, different PCR-based methods have been developed for the diagnosis of malaria, including PCR-restriction fragment length polymorphism, multiplex PCR, Nested-PCR, loop-mediated isothermal amplification, quantitative real-time PCR (qPCR), and more recently, droplet digital PCR (ddPCR) (Snounou et al., 1993; Perandin et al., 2004; Mangold et al., 2005; Han et al., 2007; Lucchi et al., 2010; Koepfli et al., 2016; Srisutham et al., 2017). Furthermore, there are many efforts for the development of molecular diagnostic methods for detection of other *Plasmodium* species that presents a challenge for species identification by LM, such as *P. malariae, P. ovale*, and the zoonotic malaria parasites*, P. knowlesi* and *P. simium* (Piera et al., 2017; Srisutham et al., 2017; de Alvarenga et al., 2018).

The small subunit of the ribosomal RNA gene (18S rRNA), which has 4–8 copies in the genome, is the most commonly used target for malaria diagnosis (Mercereau-Puijalon et al., 2002). In recent years, increasingly sensitive PCR methods have emerged targeting multicopy genes in the parasite genome (Lucchi et al., 2013; Hofmann et al., 2015; Lloyd et al., 2018). Studies on malaria diagnosis have shown that mitochondrial DNA amplification (mtDNA) results in high sensitivity due to the large number of copies of mtDNA (≈20 copies) (Polley et al., 2010; Gruenberg et al., 2018). Although mtDNA is a sensitive target, there is 90% conservation between *P. vivax* and *P. falciparum*, which hampers the design of specific distinguishing assays (McIntosh et al., 1998). In 2011, Demas and colleagues identified two new multicopy targets in the subtelomeric regions of *P. vivax* and *P. falciparum*: Pvr47 (14 copies) and Pfr364 (41 copies) (Demas et al., 2011). First, the protocol published by Demas was based on conventional PCR, but we recently adapted it for qPCR, which showed good sensitivity in the detection of co-infections in samples with low parasite densities and submicroscopic malaria among asymptomatic patients (Amaral et al., 2019).

Blood sampling for the diagnosis of malaria offers few risks to the patient if performed by experienced professionals following the standard criteria for collection. However, certain groups, such as indigenous people, devotees of some religions, infants, children, and pregnant women may have restrictions to collecting blood, particularly when repeated blood sample collections are required, such as for treatment control follow-up. A less invasive type of sampling, such as saliva collection, has been an option for point-of-care diagnostic methods, which are considered to be important tools for molecular diagnosis (Malamud, 2011). The use of saliva is ideal because of its practicality in the collection, transportation, and storage, in addition to being collected using buccal swabs (Virkler and Lednev, 2009; Köhnemann and Pfeiffer, 2011; Pfaffe et al., 2011). Some studies have assessed the use of saliva for molecular diagnosis by detecting 18S rRNA, mtDNA, and varATS targets (Buppan et al., 2010; Putaporntip et al., 2011; Ghayour Najafabadi et al., 2014; Mfuh et al., 2017; Lloyd et al., 2018). The sensitivity of saliva testing varied according to the species, molecular target, PCR method, and reference standard used, ranging from 74 to 84%.

To overcome the low sensitivity of molecular methods due to the low amount of parasite DNA in saliva, we developed a new method based on ddPCR for the diagnosis of malaria by using multicopy Pvr47 and Pfr364 targets. The proposed method may improve the diagnosis of malaria in endemic regions by allowing the detection of a greater number of infections characterized by low parasitemia, as well as co-infection with different *Plasmodium* species. Additionally, it has other potential applications, such as its use in reference centers for the diagnosis of malaria, especially in regions that lack experienced microscopists, and as a tool for the epidemiologic surveillance of malaria.

## Materials and Methods

### Subjects and Sample Collection

The samples assessed here were collected from two Brazilian states, Rondônia (RO) and Roraima (RR), from 2017 to 2020. Samples were colleted in the state capital, Boa Vista (RR) and Porto Velho (RO). Both cities are in the Amazon area, with a humid tropical climate, including two major seasons: a rainy season between April and November, with high rainfall indices during June and July, and a dry season between December and March.

Enrolled patients sought public health services with malaria symptoms and provided informed consent to participate in the study. The majority of the study population were adults with a median age of 36 years (interquartile range, 26.5–45 years) and a female:male ratio of 1:3. *Plasmodium* spp. infection was confirmed via optical microscopy based on Giemsa-stained thick blood smears evaluated by well-trained microscopists, in accordance with the malaria diagnosis guidelines of the Brazilian Ministry of Health. Parasite density was determined as the number of asexual parasites observed per 200 white blood cells on a thick smear and was estimated by assuming a leukocyte count of 8,000 per μL. A total of 471 samples were included in this study (157 each of blood, saliva, and buccal swab samples). Because we had limited amounts of DNA of each sample, many samples could not be assessed using the two amplification methods (qPCR and ddPCR). Additionally, 30 healthy uninfected human volunteers from a malaria-free area (Belo Horizonte, Minas Gerais, Brazil) served as negative controls. For saliva and buccal swab sample collection, patients were required not to ingest any kind of food or drink 30 min before the procedure. At least 1 mL of saliva was collected from each patient. For swab collection, the brush was inserted into one side of the mouth and repeatedly twisted against the inner cheek until saturation. Both samples were collected without preservatives and stored at −20°C until they were transferred to the René Rachou Institute, where they were maintained at −80°C until DNA extraction.

The ethical and methodological aspects of this study were approved by the Ethical Committee of Research on Human Beings of the René Rachou Institute (CAAE 70755617.8.0000.5091) according to the Brazilian National Council of Health (Resolutions 196/96 and 466/12). All adult participants signed written informed consent forms, whereas next of kin, caretakers, or guardians signed on behalf of the minors/children enrolled in the study. All methods were carried out following the approved guidelines.

### Extraction of Genomic DNA

DNA samples were extracted from 300 μL of peripheral blood collected in EDTA-containing tubes and from 1 mL of saliva for parasite genomic analysis using the Gentra Puregene Blood Kit (QIAGEN, Chatsworth, CA, USA) according to the manufacturer's instructions. Extracted DNA from the blood and saliva was resuspended in 50 μL of hydration solution. DNA from swabs was extracted using the Purelink Genomic DNA mini kit (Thermo Fisher Scientific, Waltham, MA, USA) and resuspended in 30 μL elution buffer. In the preparation of swab lysates, the following modifications were made: 200 μL of 1X phosphate buffered saline was added to each sample during the first step of the protocol and was incubated at 55°C for 30 min instead of 10 min.

### Standard Curves to Estimate qPCR Efficiency, Limit of Detection, and Quantification of Parasitemia

Standard curves were generated with linearized plasmids by restriction digestion containing the target sequences, and the efficiency of the qPCR was calculated (Supplementary Material). To generate standard curves, five-fold serial dilutions were prepared ranging from 2.0 × 10^4^ to 5.12 × 10^−2^ copies/μL and run in triplicate.

Clinical blood samples from *P. vivax-* and *P. falciparum*-infected patients with parasite densities confirmed by an expert microscopist were used to determine the limit of detection of assays as well as estimate parasite densities using qPCR and ddPCR. For each species, three-fold serial dilutions were prepared, ranging from 220 to 0.3 parasites/μL. qPCR was performed in triplicate (for high concentrations) or quintuplicate (for low concentrations), whereas ddPCR was performed in triplicate for all concentrations. For the quantification of parasitemia via qPCR (in parasites/μL), parasite densities were adjusted according to the concentration factor (1.2-fold) of the DNA template based on the blood volume equivalent of 12 μL used in PCR reactions. The blood volume equivalent considers the concentration factor of the DNA 6-fold during nucleic acid extraction, that is, resuspension of the DNA in 50 from 300 μL of blood multiplied by the volume of DNA (2 μL) added to the reactions.

### *Plasmodium vivax* and *P. falciparum* Amplification *via* qPCR

The amplification of the Pvr47 and Pfr364 genes was conducted using a previously described protocol (Amaral et al., 2019). To increase the signal detection and sensitivity of the *P. falciparum* assay, modifications were introduced in the length of the probe and both primers. The set of oligonucleotides for Pvr47 was 5′ TCCGCAGCTCACAAATGTTC 3′ (forward), 5′ ACATGGGGATTCTAAGCCAATTTA 3′ (reverse), and 5′- FAM-TCCGCGAGG-ZEN-GCTGCAA-Iowa Black FQ 3 (probe). The primers used for Pfr364 were 5′ CTCGCAATAACGCTGCAT 3′ (forward), 5′ TTCCCTGCCCAAAAACG 3′ (reverse), and 5′ FAM-TGGTGCCGG-ZEN-GGGTTTCTACGC-Iowa Black FQ 3′ (probe). The reactions were performed in 10 μL volumes containing 2 μL of DNA (approximately 50 ng) and 5 μL of TaqMan Universal PCR Master Mix (Thermo Fisher Scientific). For the Pvr47 amplification, 50 nM of forward primer, 900 nM of reverse primer, and 250 nM of probe were used; for Pfr364, 900 nM of forward primer, 300 nM of reverse primer, and 150 nM of probe were used. The qPCR assays were performed using the ViiA7 Real-Time PCR System (Thermo Fisher Scientific), with the following cycling parameters: pre-incubation and initial denaturation at 50°C for 2 min and 95°C for 10 min, followed by 40 cycles of denaturation at 95°C for 15 s, primer annealing at 52°C for 1 min, and extension at 60°C for 1 min. Fluorescence was verified at the end of each extension step. qPCR was repeated whenever discordant results were obtained among blood, saliva, and swab samples.

### Development and Validation of the Droplet Digital PCR Assays

The ddPCR assays were prepared using the same primers and probes used in qPCR, with a total of 22 μL per reaction containing ddPCR reagents (10 μL of the Bio-Rad 1X ddPCR Super Mix [no dUTP], 900 nM of forward and reverse primers, and 250 nM of the probe), and 2 μL of the DNA template. Initially ddPCR was carried out in a Bio-Rad QX200TM Droplet Generator. Later, a QX200 AutoDG Droplet Digital PCR System was used to automatically generate droplets. To optimize the PCR annealing temperature, a temperature gradient of 57–59°C was used. An annealing temperature of 58°C provided better separation between the positive and negative droplets for both *P. vivax* and *P. falciparum* (data not shown). Endpoint PCR assays were performed using the following cycling parameters: enzyme activation at 95°C for 5 min, followed by 40 cycles of denaturation at 94°C for 30 s, and primer annealing at 58°C for 1 min. The results were analyzed using a Bio-Rad QX200TM Droplet Reader. Additionally, a false-positive cutoff for ddPCR assays was established by calculating the limit of blank (LoB). To do so, we used negative controls for both *Plasmodium* species, that is, all the reactions without the DNA template for the independent assays. A threshold to promote a better separation of low fluorescence amplitude droplets was determined to be 3,000 and 4,000 RFU, respectively, for *P. vivax* and *P. falciparum* assays. ddPCR was repeated whenever discordant results were obtained among blood, saliva, and swab samples. Target quantification was expressed as copies/μL of the ddPCR reaction, that is, without any conversion. Wherever indicated, the results were expressed as copies/μL of blood following the formula: target copies/μL of blood = [(copies/μL from ddPCR × 22 (total volume of ddPCR in μL)]/[2 (total loaded DNA in μL) × 6 (6X concentrated DNA)] (Srisutham et al., 2017). All samples that were negative for the diagnostic reactions (qPCR and ddPCR) were subjected to a PCR assay for the amplification of a constitutive gene (ABO blood group) using primers that we previously described and adapted for qPCR (Olsson et al., 1998; Robortella et al., 2020).

### Statistical Analysis

Continuous variables were compared using the Mann–Whitney test or Kruskal–Wallis test with Dunn's *post-hoc* test, as appropriate. Proportions are given with 95% confidence intervals and were compared using the χ^2^ test, Fisher's exact test, or McNemar's test (paired data). Statistical analysis was performed using the R v.4.1.1 package, STATA v.14 software, and GraphPad Prism version 8.0.2 (GraphPad Software, San Diego, California, USA). Accuracy measures of diagnostic tests (sensitivity and specificity) were estimated using the *Forest* function available in the R package DTAplots. To estimate the sensitivity and specificity of each protocol, we defined the combined results of all qPCR (or ddPCRs) runs as a reference, excluding those from the protocol under evaluation (Hofmann et al., 2018).

## Results

### Performance of qPCR With Different Sources of DNA

**Figure 1:**
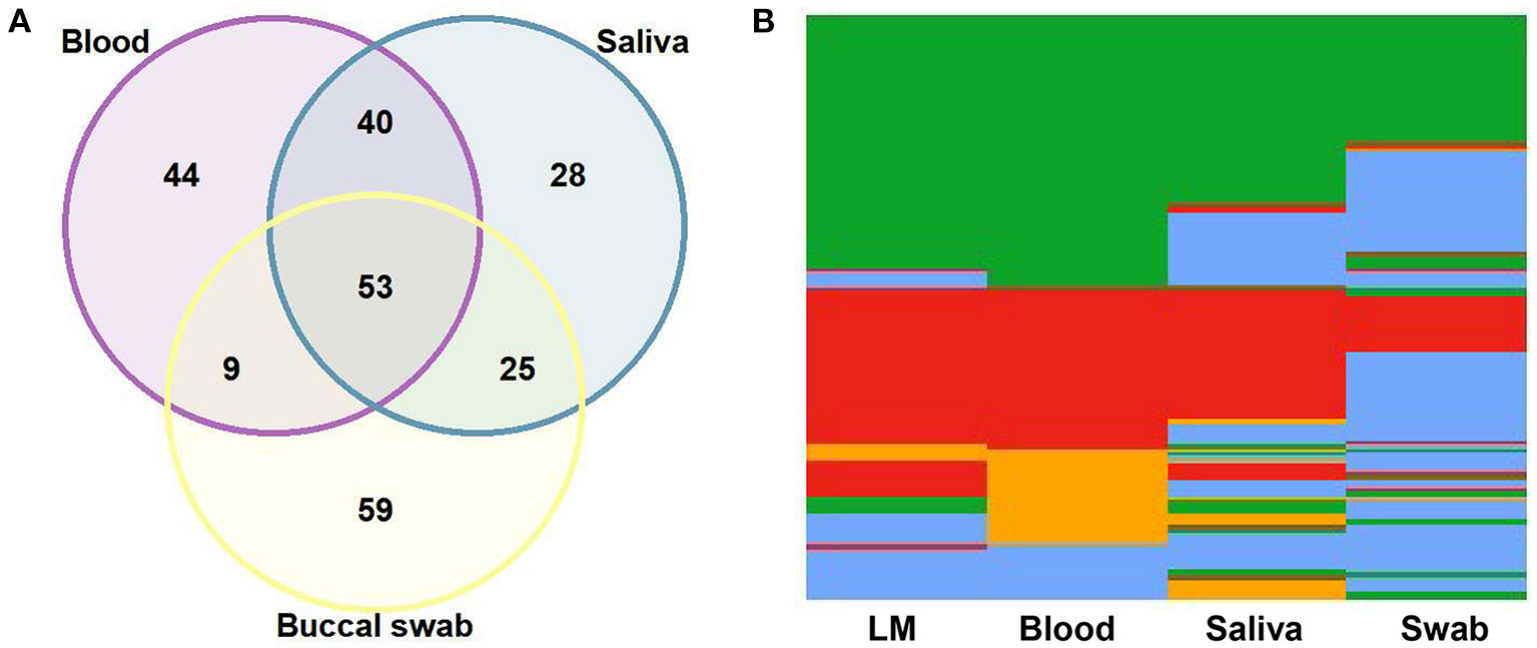
Comparison of quantitative real-time PCR (qPCR) results among 146 paired samples of blood, saliva, and buccal swabs. **(A)** Venn diagram showing the agreement (in absolute number) among blood, saliva, and swab samples. **(B)** Heatmap for the results of light microscopy (LM) and qPCR of blood, saliva, and swab samples: *Plasmodium vivax*, in green; *P. falciparum*, in red; mixed-species infections, in orange; negative samples, in blue.

To evaluate the performance of qPCR in detecting parasite DNA in the different DNA sources, 146 paired samples from blood, saliva, and buccal swabs were analyzed. qPCR of saliva from those with blood-positive samples amplified 71% of *P. vivax* and 82% of *P. falciparum* (Supplementary Table 2). Identical results were obtained for 93 (64%) blood and saliva samples, 62 (42%) blood and swab samples, and 78 (53%) saliva and swab samples (Figure 1A). Most swab samples failed to show DNA amplification via qPCR, even though all swab samples with negative results successfully amplified the control in the human chromosome (ABO blood group). Many mixed-species infections were exclusively detected in the blood and saliva using qPCR (Figure 1B).

**Figure 2:**
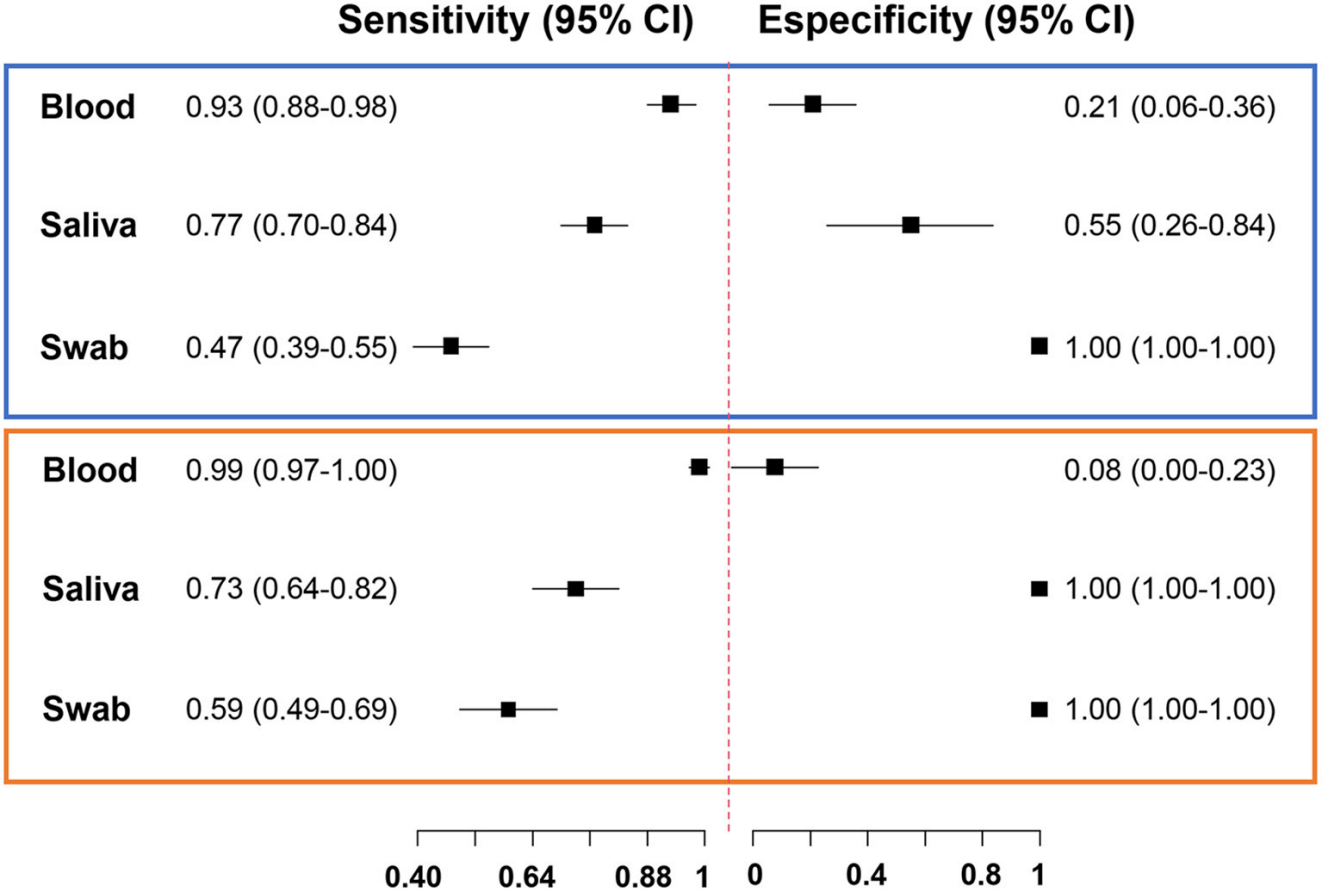
Forest plot of accuracy measures of diagnostic tests. Sensitivity and specificity of quantitative real-time PCR (chart in blue) and droplet digital PCR (chart in orange) results of blood, saliva, and swab samples.

Next, we estimated the accuracy measures of the diagnostic tests for the subset of 146 paired samples. The reference method for each protocol was defined by combining the results of all qPCR and excluding the protocol under evaluation. Thus, the sensitivity of qPCR in blood was 93%, followed by 77% in saliva, and 47% in swabs (Figure 2; Supplementary Table 3). qPCR in blood had low specificity, as expected, indicating that the method has a lower detection threshold (higher positivity). None of the healthy uninfected human blood samples showed any amplification of *Plasmodium* via qPCR.

**Figure 3:**
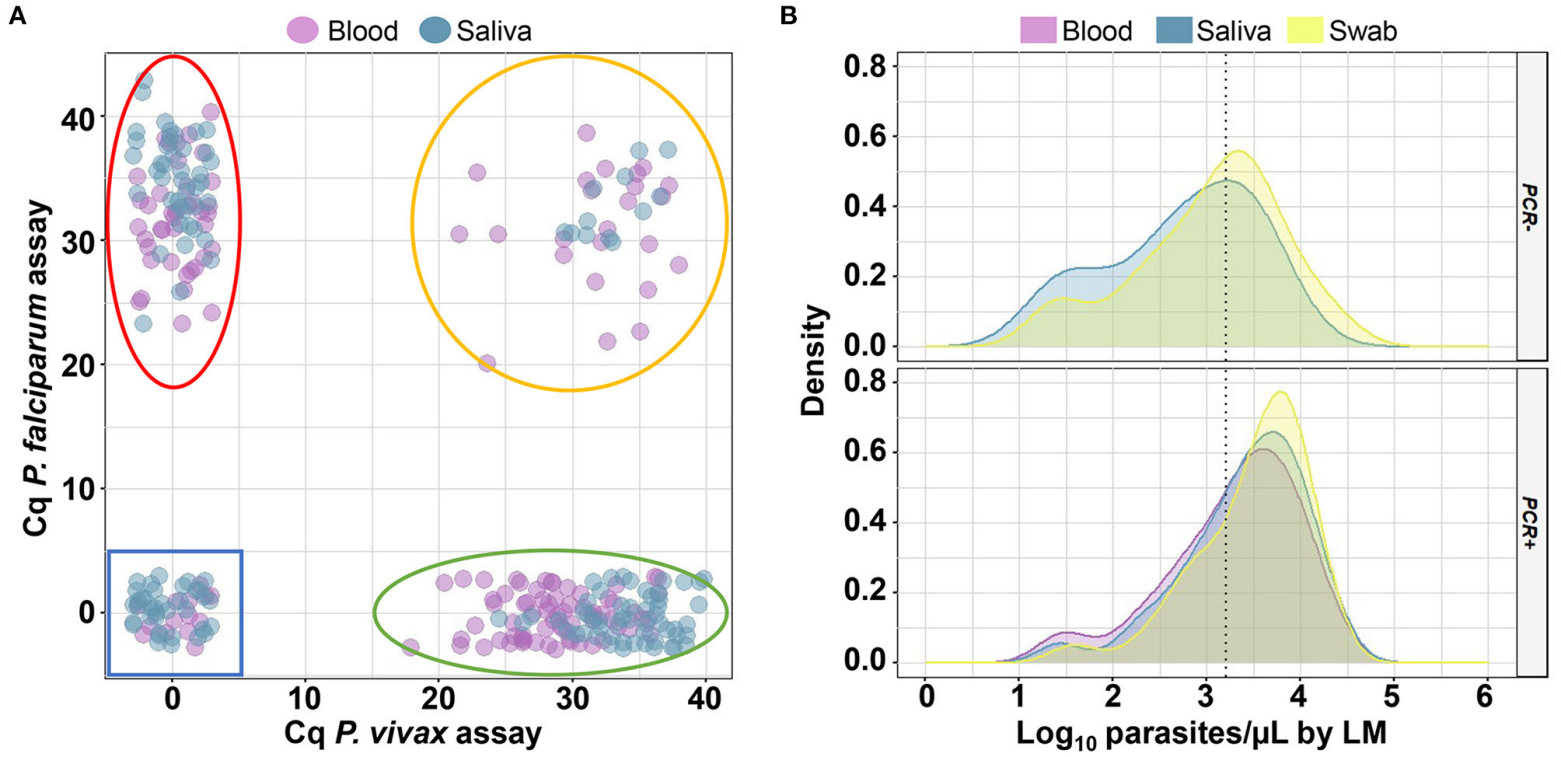
Analysis of quantitative cycles (Cq) and parasitemia for samples analyzed via quantitative real-time PCR (qPCR). **(A)** Scatter plot of Cq measured via qPCR for individual samples (small circles) of blood and saliva. Delimited regions in the plots indicate where *Plasmodium falciparum* (red line), *P. vivax* (green line), mixed-species infections (orange line), and negative samples (blue line) should be located. **(B)** Density plot showing the distribution of log_10_ of parasite density estimated through microscopy for 82 samples which were not amplified (PCR -) and 272 samples which were amplified (PCR +) via qPCR in the blood (purple curves), saliva (blue curves), and buccal swab (yellow curves) samples. The dotted line indicates a parasite density of 1,500 parasites/μL (Log_10_ = 3.18). This analysis was performed for 118 paired samples with known parasitemia.

There was a tendency for qPCR-positive saliva samples to show higher Cq values than blood (Figure 3A). Notably, the mean Cq value of saliva samples did not differ, regardless of the storage time of the saliva (Supplementary Table 4). Considering only the samples with known parasitemia by LM, 82 out of 354 (23%) samples from different sources of DNA (1 sample of blood, 24 of saliva, and 57 of swab) failed to amplify the parasite target via qPCR. Among them, saliva did not amplify parasite DNA, mostly in samples from patients with low-grade infections (67%, <1,500 parasites/μL) (Figure 3B). For swabs, a lower proportion of negative results (45%) could be explained by low parasitemia.

### Performance of ddPCR With Different Sources of DNA

In ddPCR, the limit of detection of the Pvr47 and Pfr364 assays was estimated using clinical samples with known parasitemia. The limit of detection in blood samples fluctuated for both assays but was consistently below 1 parasite/μL for *P. falciparum* (0.1–0.9 parasites/μL for *P. falciparum* and 0.9–2.7 parasites/μL for *P. vivax*) (Supplementary Table 5). In addition, a positive droplet cutoff (LoB) was established, that is, the results were considered positive for three positive droplets. The reproducibility and repeatability of ddPCR quantification were high, even at low parasite densities (Supplementary Figures 1C,D).

**Figure 4:**
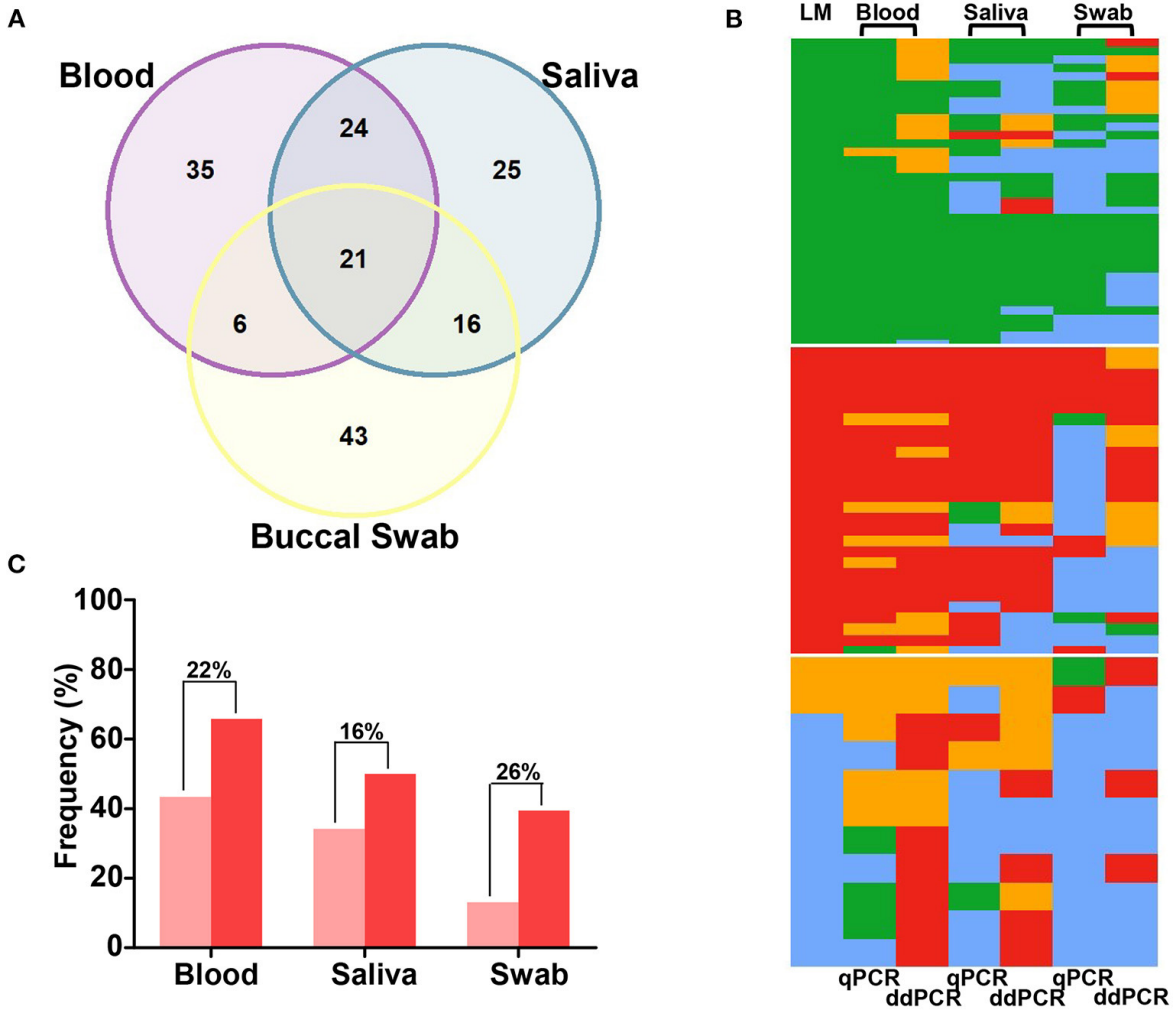
Comparison of quantitative real-time PCR (qPCR) and droplet digital PCR (ddPCR) results for paired samples of blood, saliva, and buccal swabs. **(A)** Venn diagram showing the agreement (in absolute number) in ddPCR for 86 paired samples of blood, saliva, and swabs. **(B)** Heatmaps for qPCR and ddPCR results of 76 paired-samples of blood, saliva, and swabs: *Plasmodium vivax*, in green; *P. falciparum*, in red; mixed-species infections, in orange; negative samples, in blue. Data were grouped based on light microscopy (LM) results (37 *P. vivax* infections, 28 *P. falciparum* infections, and 11 mixed or negative infections). **(C)** The proportion of *P. falciparum* infections (single or mixed infections) detected via qPCR (light red) and ddPCR (dark red). Differences in proportions are indicated above bars.

We evaluated the performance of ddPCR for malaria detection in 86 paired blood, saliva, and swab samples. ddPCR of saliva amplified 57% of *P. vivax* and 76% of *P. falciparum* in ddPCR-positive blood samples (Supplementary Table 6). Overall, concordant results were obtained for 52% (*n* = 45) of blood and saliva samples, 31% (*n* = 27) of blood and swab samples, and 43% (*n* = 37) of saliva and swab samples via ddPCR (Figure 4A). The sensitivity of ddPCR in blood was 99%, whereas saliva and swabs showed sensitivities of 73% and 59%, respectively (Figure 2; Supplementary Table 7). Here, the specificity of ddPCR should be interpreted with caution because of the small number of true-negative samples analyzed. Notably, 30 healthy uninfected human blood samples were tested, and none showed amplification when assessed via ddPCR with either assay.

Among all samples that showed no amplification via ddPCR and had known parasitemia via LM, only a portion (39%, 20 out of 51) were from patients with low-grade infections (<1,500 parasites/μL). There was no significant association between the storage time of the samples and the number of copies/μL estimated in saliva via ddPCR (Supplementary Table 8).

### Comparison of Molecular Diagnostic Methods: qPCR and ddPCR

A subset of 76 samples was evaluated using two molecular tests with different sources of DNA. Overall, a similar proportion of *Plasmodium* infection was detected via qPCR and ddPCR in the blood (73 and 75, respectively) and saliva (55 and 57, respectively). However, ddPCR detected 16% more infections in swabs (*n* = 46, 61%) than qPCR (*n* = 34, 45%) (*P* = 0.045 by McNemar's test) (Table 1; Figure 4B). Moreover, a higher number of mixed infections were detected via ddPCR in the blood (22 and 11 by ddPCR and qPCR, respectively, *P* = 0.0098 by McNemar's test), saliva (9 by ddPCR and 2 by qPCR, *P* = 0.0233 by McNemar's test), and swabs (14 by ddPCR and 0 by qPCR, *P* = 0.0005 by McNemar's test). In other words, the proportions of mixed infections not detected by qPCR were 15%, 9%, and 18% in the blood, saliva, and swab samples, respectively. In general, the differences between the two molecular tests were due to the detection of a higher number of *P. falciparum* infections using ddPCR (Figure 4C).

**Table 1:** Summary of results of quantitative real-time PCR (qPCR) and droplet digital PCR (ddPCR) for 76 paired samples of blood, saliva, and swab.

	**N°of samples (%)**					
	**qPCR Blood**	**ddPCR Blood**	**qPCR Saliva**	**ddPCR Saliva**	**qPCR Swab**	**ddPCR Swab**
*P. vivax*	40 (52.6)	25 (32.9)	29 (38.2)	19 (25.0)	24 (31.6)	16 (21.1)
*P. falciparum*	22 (28.9)	28 (36.8)	24 (31.6)	29 (38.2)	10 (13.1)	16 (21.1)
Mixed^a^	11 (14.5)	22 (29.0)	2 (2.6)	9 (11.8)	0 (0.0)	14 (18.4)
Negative	3 (4.0)	1 (1.3)	21 (27.6)	19 (25.0)	42 (55.3)	30 (39.5)
Total Positivity	73 (96.0)	75 (98.7)	55 (72.4)	57 (75.0)	34 (44.7)	46 (60.5)

a *Mixed-species infection (P. vivax/P. falciparum)*.

### Agreement Between Parasite Density Estimates

**Figure 5:**
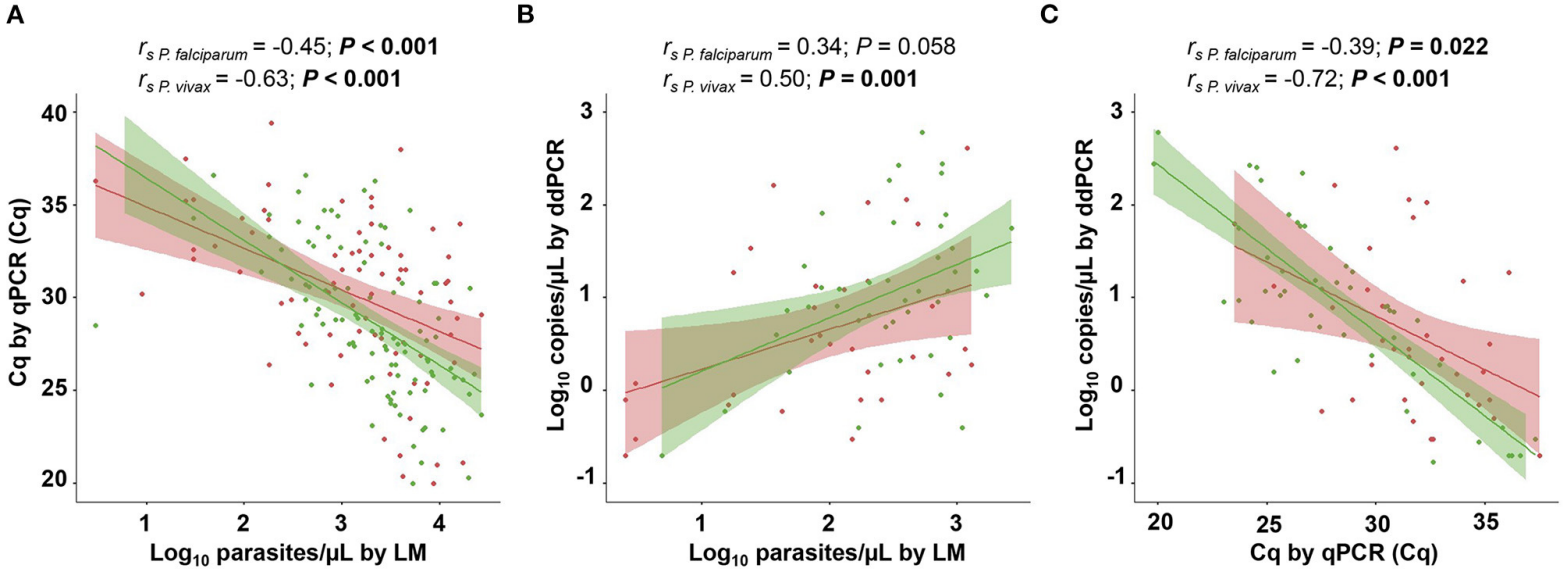
Correlation analysis between parasite densities estimated via light microscopy (LM) and molecular diagnostic methods. **(A)** Parasitemia levels determined via LM (parasites/μL) vs. quantitative cycles (Cq) measured using quantitative real-time PCR (qPCR). **(B)** Parasitemia levels determined through LM and droplet digital PCR (ddPCR) (copies/μL). **(C)** Cq measured through qPCR vs. parasitemia levels determined via ddPCR. Spearman correlation coefficient was calculated independently for *Plasmodium vivax*-positive (green) and *P. falciparum*-positive samples (red). *P* values of <0.05 are shown in boldface. Parasitemia levels estimated via LM and ddPCR are presented as log_10_. The shaded area around the regression line represents the 95% confidence interval (CI).

We evaluated the level of agreement of density estimates between the different methods in blood, which is the major site of infection. Initially, the Cq values obtained via qPCR were compared to the density estimates obtained via LM (parasites/μL) and ddPCR (copies/μL). Overall, there was a moderate correlation between parasite densities estimated by different methods in the blood. For *P. vivax*, a significant correlation was observed for all three analyses (Figures 5A–C). For *P. falciparum*, the density estimates obtained via ddPCR and LM did not correlate with each other (Figure 5B). The geometric mean densities estimated via ddPCR was 11.5 copies/μL (95% CI, 6.1–21.8 copies/μL) and 4.1 copies/μL (95% CI, 1.8–9.4 copies/μL), respectively, for *P. vivax* and *P. falciparum*. By converting in copies/μL of blood, the geometric mean density was 21.0 copies/μL (95% CI, 11.1–40.0 copies/μL) of blood for *P. vivax* and 7.6 copies/μL (95% CI, 3.4–17.3 copies/μL) of blood for *P. falciparum*.

**Figure 6:**
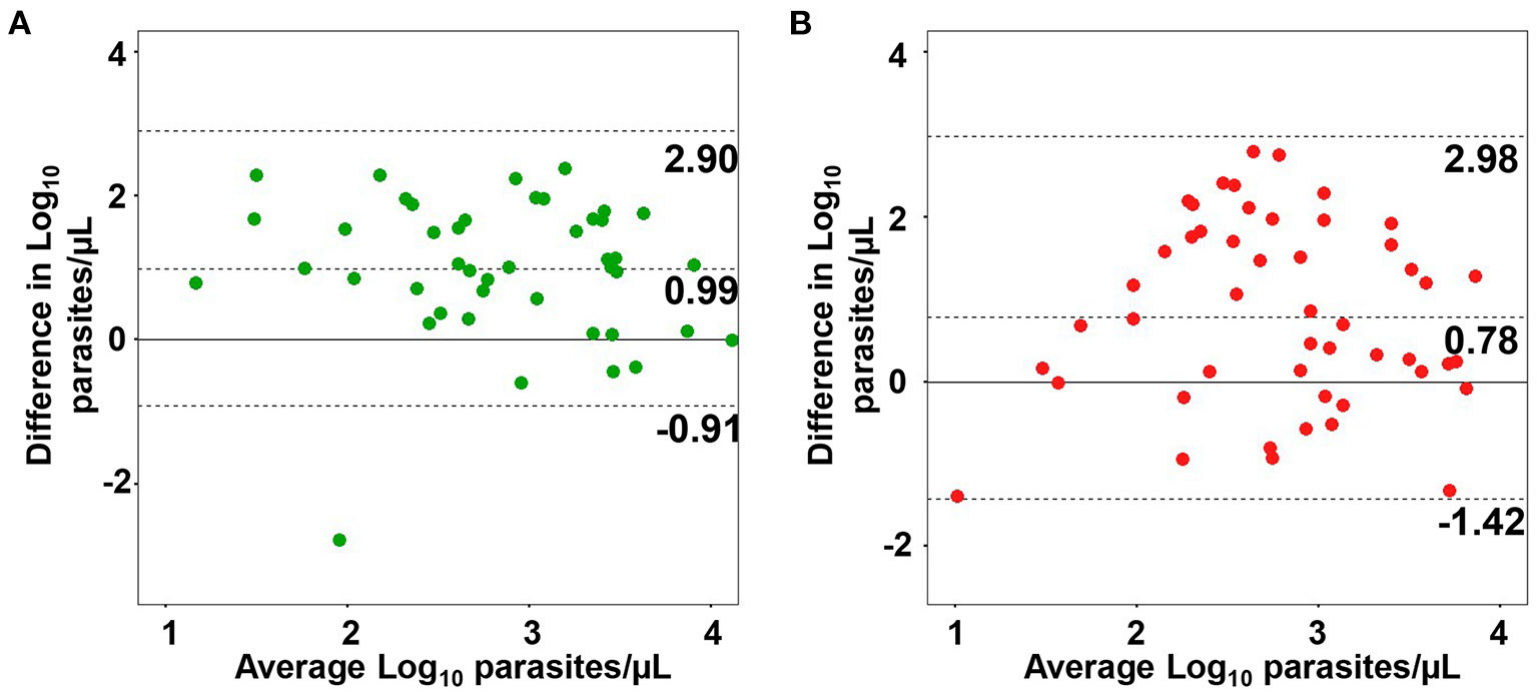
Agreement between parasite densities estimated via microscopy (LM) and quantitative real-time PCR (qPCR). Bland–Altman plots of agreement between parasite density in blood estimated via LM and qPCR for *Plasmodium vivax* **(A)** and *P. falciparum* samples **(B)**. The dotted middle line and two-outer dotted lines represent the 95% region of agreement.

Considering that there was a significant correlation between qPCR and LM for both species, we obtained the parasite densities via qPCR, as estimated through microscopy (parasite/μL). Parasite densities were determined for 44 clinical samples of *P. vivax* and 52 samples of *P. falciparum* by applying a linear regression equation (Supplementary Material). Concordance analysis was performed to evaluate the degree of agreement of parasite density estimates between LM and qPCR in the blood. The differences between estimates obtained via LM and qPCR in blood were evenly scattered, showing similar agreements across the whole range of parasitemia levels (Figures 6A,B). The mean difference was positive and statistically significant for both species, 0.99 log units (95% IC, 0.70–1.29, *P* < 0.001 by paired *t*-test) for *P. vivax* and 0.78 log units (95% CI, 0.46–1.09, *P* < 0.001 by paired *t*-test) for *P. falciparum*, implying that the estimates of parasite density via LM are higher than qPCR estimates. Hence, these values can be used to adjust blood parasitemia estimates assessed via qPCR. The Bland–Altman plot for *P. falciparum* demonstrated a higher dispersion in estimates obtained via LM and qPCR, as observed by the distance between the two limits of agreement (2.98, −1.39), indicating less agreement with microscopy counts for this species.

## Discussion

One of the priorities of malaria research is the development of alternative assays for the diagnosis of *Plasmodium* spp. infections that are highly sensitive, specific, and minimally invasive. It is estimated that more than 50% of malaria cases are not detected using microscopy (Okell et al., 2009). Thus, it is essential to develop new diagnostic methods that can detect low-density infections for successful malaria control and elimination. Additionally, the use of non-invasive specimens as the DNA source offers many advantages for patients who have some restriction for blood collection or who require repeated sampling or testing for *Plasmodium* infection (Wright et al., 2009).

Here, we evaluated the performance of molecular diagnostic assays based on qPCR and ddPCR amplification of multicopy targets (Pvr47/Pfr364) in the *Plasmodium* genome to detect malarial parasites in saliva and buccal swabs. Except for mixed-species infections, there was good concordance between qPCR results in saliva and blood. A higher concordance was observed for *P. falciparum* detection in saliva, which amplified 82% of *P. falciparum* and 71% of *P. vivax* qPCR-positive samples in the blood. The *P. falciparum* assay targeting a higher number of gene copies in the parasite genome (Demas et al., 2011) could explain the higher sensitivity of the method to detect this species via qPCR in saliva. In contrast to saliva, in swabs and blood, the concordance between qPCR results was fairly low. While the sensitivity of qPCR in saliva was 77%, it was only 47% for swabs. As previously shown, the sensitivity of malaria parasite detection depends on the volume of the sample analyzed (Hofmann et al., 2018). Our protocol included the screening of 1 mL of saliva, which may significantly improve the sensitivity of the method over swabs. Our analysis showed that parasite DNA could not be detected in saliva, mainly in low-density infections (<1,500 parasites/μL). The association between the level of parasitemia and parasite detection was less clear for swabs. We also raised the possibility that the storage conditions of the samples could interfere with the performance of diagnostic tests. Indeed, studies have shown that the quality of genomic DNA obtained from saliva can be affected by the collection, storage conditions, and DNA extraction protocol (Buppan et al., 2010; Garbieri et al., 2017). The samples analyzed were frozen (−20 and −80°C) for different periods (1–32 months) until they were processed for DNA extraction. However, our analysis suggests that storage time did not significantly interfere with the diagnostic test results. Furthermore, it has been demonstrated that the use of commercially available kits with reagents that stabilize the saliva sample before DNA extraction may increase the overall sensitivity of the method (Garbieri et al., 2017). The evaluation of different protocols of collection/storage was beyond the scope of the present study, but we consider that some adjustments in the procedure of sample collection may improve the performance of our tests, particularly for buccal swabs. Further studies should consider the impact of oral health on the accuracy of malaria diagnostic tests because conditions that cause gingival bleeding or oral inflammation could contribute to an increase in blood derivatives in saliva.

To explore amplification protocols that use alternative sources of parasite DNA, we developed a new diagnostic method based on ddPCR. In general, ddPCR showed increased sensitivity for detecting *Plasmodium* (99% in blood, 73% in saliva, and 59% in swabs) compared with qPCR. Because of the limited number of negative paired samples of blood, saliva, and swabs, specificity should be interpreted with caution, and it remains to be estimated in a larger study. Interestingly, the ddPCR diagnostic test results were less concordant between the sources of parasite DNA assessed. This discrepancy could be explained in part by the misdiagnosis of *P. vivax* as a mono- or mixed infection, probably due to the lower number of Pvr47 copies. Although the total positivity did not differ between ddPCR and qPCR in the blood and saliva, ddPCR detected a higher number of infections in swabs. ddPCR also revealed a significantly higher number of *P. vivax*/*P. falciparum* mixed infections from different DNA sources. Surprisingly, we found a higher proportion of mixed infections in swabs than in saliva using ddPCR. Our data suggest that the differences between ddPCR and qPCR were the result of a higher number of *P. falciparum* infections detected via ddPCR. We have raised some possibilities to explain these findings. First, it follows that the higher number of gene copies targeted by the *P. falciparum* assay has a greater impact on assay sensitivity in ddPCR than in qPCR. Second, some studies described ddPCR as more resistant to PCR inhibitors frequently present in some types of samples, such as blood (Dingle et al., 2013; Taylor et al., 2017). In addition, ddPCR is more likely to detect low-concentration targets with high reproducibility when compared to qPCR, and this feature remains even in different types of samples, as recently shown for the detection of *P. falciparum* in serum (Hindson et al., 2013; Pomari et al., 2020). We believe that ddPCR features contributed to better performances in saliva and swabs. Finally, a large proportion of *P. falciparum* is sequestered in the deep vasculature of internal organs, and its density in the blood can be often underestimated (Franke-Fayard et al., 2010). Here, we cannot exclude the possibility that parasite DNA could reach other fluids in the body without a direct correlation with what is found in the blood (Buppan et al., 2010; Mfuh et al., 2017).

Molecular diagnostic approaches can also readily provide a robust quantification of malarial parasites, overcoming the limitations of LM in detecting and quantifying low-density infections. Quantitative real-time PCR can be easily employed to estimate parasite densities; thus, we compared the estimates obtained via LM and qPCR in the blood. There was a moderate correlation between LM counts and qPCR parasite density estimates, with LM providing higher parasite count estimates. An overestimation of parasite counts using LM has also been observed in previous reports (Rougemont et al., 2004; Nwakanma et al., 2009). In the present study, the analysis of the Bland–Altman plot showed an agreement for parasite density estimates between LM and qPCR in blood. This agreement was slightly higher for parasite densities obtained for *P. vivax*. Similarly, there was no correlation between the estimates of parasitemia for *P. falciparum* obtained via LM and ddPCR in the blood. In principle, we believe that the significant difference in sensitivity between these methods may have influenced our results. We cannot exclude the possibility of inaccurate parasite density estimates using LM. Furthermore, the sequestration of *P. falciparum* in the deep capillaries, or even the loss of DNA during the extraction procedure, could lead to an underestimation of parasite density. An interesting result was obtained by Koepfli et al. (2016), who reported more than 50% loss of DNA during extraction. Despite the loss of DNA, there was a correspondence between copies/μL obtained via ddPCR by targeting the 18S RNA gene and parasites/μL counted via LM. In contrast, we found that the parasitemia estimates via ddPCR and qPCR were more correlated with each other for both species. Likewise, a high correlation in quantification by ddPCR and qPCR but only a moderate correlation between ddPCR and LM have been previously described (Koepfli et al., 2016). Because DNA quantification by qPCR depends on a standard curve, which is costly and time-consuming and may not be reliable in common situations when the amplification product is above 30 cycles, ready-to-go quantification through ddPCR may be crucial for malaria treatment with parasite DNA measurement as an indicator of the disease outcome (Karlen et al., 2007; Imwong et al., 2015).

Altogether, our findings on submicroscopic *P. falciparum* and mixed infections may significantly affect efforts to eliminate malaria in the Amazon region. This is particularly important in Brazil, where the proportion of *P. falciparum* cases has increased by 32.6% in 2020 compared to 2019 (BRASIL. BRASIL Ministério da Saúde 2021). Malaria treatment depends on the correct identification of *Plasmodium* species and misdiagnosis may pose a risk to individual health. However, control measures should consider the parasite species that circulate in the area. Recently, a systematic review showed a high risk of *P. vivax* malaria recurrence due to hypnozoite reactivation after *P. falciparum* malaria treatment (Commons et al., 2019). The commonly recommended therapy for *P. falciparum* malaria includes artemisinin-based combination therapies (ACTs), which have high efficacy against malarial blood stages but not against liver hypnozoites. This highlights the importance of identifying parasite species for the appropriate clinical management of malaria. One limitation of the present study is that the patients were not followed up; thus, data on malaria recurrence after treatment were not available. These data could provide a better picture of the consequences of misdiagnosing low-density infections. An important issue addressed recently was the diagnostic sensitivity necessary to guide malaria interventions (Hofmann et al., 2018). This study analyzed large blood volumes via ultra-sensitive qPCR (us-qPCR) and found a large pool of ultra-low-density *P. vivax* and *P. falciparum* infections (Hofmann et al., 2018). The performance of us-qPCR was assessed in areas of low to moderate transmission intensity, showing substantial gains in the detection of *P. vivax* and *P. falciparum* over the standard qPCR (Gruenberg et al., 2020). Whether these infections are infective remains to be determined, as gametocyte densities were found to be very low (Kiattibutr et al., 2017).

## Conclusions

The molecular diagnosis of malaria using the multicopy targets Pvr47 and Pfr364 through highly sensitive detection methods, such as ddPCR, proved to be useful for the detection of *P. falciparum* and *P. vivax* infections. We found that ddPCR outperformed qPCR for the differentiation between *P. falciparum* mono-infections and mixed-species infections. This result reinforces our previous finding that qPCR assays targeting Pvr47/Pfr364 are sensitive in detecting low levels of *P. vivax*/*P. falciparum* mixed infections (Amaral et al., 2019). This improvement in the diagnostic method has a direct impact on treatment and disease outcomes. Furthermore, the detection of parasite DNA in saliva reinforces its potential use in malaria diagnosis.

## Data Availability Statement

The original contributions presented in the study are included in the article/Supplementary Material, further inquiries can be directed to the corresponding author.

## Ethics Statement

The studies involving human participants were reviewed and approved by Ethical Committee of Research on Human Beings of the René Rachou Institute. Written informed consent to participate in this study was provided by the participants' legal guardian/next of kin.

## Author Contributions

TN was the principal investigator. GC and DA performed the qPCR analysis. GC, TO, and AF performed ddPCR analysis. TN, GC, AA, JL, and DP helped carry out the field work. TN, CF, LC, DA, and GC participated in interpretation of the data and critical revisions of the manuscript. All authors gave their final approval for publication of the manuscript.

## Funding

This study was funded by the Royal Society of Tropical Medicine and Hygiene (RSTMH Small Grants 2016), Fundação de Amparo à Pesquisa do Estado de Minas Gerais (FAPEMIG), Conselho Nacional de Desenvolvimento Científico e Tecnológico (CNPq), Programa para Inserção de Recém-Doutores nos Programas de Pós-Graduação da Fiocruz and the Programa PrInt-Fiocruz-CAPES. TN, LC, and CF are CNPq Research Productivity fellows. GC thanks the FAPEMIG for scholarship support. The funders had no role in study design, data collection and interpretation, or the decision to submit the work for publication. This study was partially supported by the Coordenaçã3o de Aperfeiçoamento de Pessoal de Nível Superior– CAPES - Finance Code 001.

## Conflict of Interest

The reviewer JL-J declared a shared affiliation with the authors GC, DA, LC, CF, TN at the time of the review. The remaining authors declare that the research was conducted in the absence of any commercial or financial relationships that could be construed as a potential conflict of interest.

## Publisher's Note

All claims expressed in this article are solely those of the authors and do not necessarily represent those of their affiliated organizations, or those of the publisher, the editors and the reviewers. Any product that may be evaluated in this article, or claim that may be made by its manufacturer, is not guaranteed or endorsed by the publisher.
