# Supplementary material for: Improving the Molecular Diagnosis of Malaria: Droplet Digital PCR-Based Method Using Saliva as a DNA Source
Source: Front Microbiol. 2022 May 13;13:882530. doi: 10.3389/fmicb.2022.882530 (PMC9136408; doi:10.3389/fmicb.2022.882530)
Supplement: Supplementary file 1 [file Data_Sheet_1.PDF]

Supplementary Material

Efficiency and limit of detection of *P. vivax* and *P. falciparum* assays in qPCR

The slope of linearized plasmid DNA curves were used to evaluate the efficiency of the *P. vivax* (Pvr47) and *P. falciparum* (Pfr364) assays in qPCR. *Plasmodium vivax* assay performed with an efficiency of 92% (R^2 = 0.998) and *P. falciparum* assay showed an efficiency of 90% (R^2 = 0.995). The limit of detection of both assays in qPCR was calculated using clinical samples with parasitemia quantified by optical microscopy. Less than 1 parasite/µL was detected by qPCR for *P. vivax* assay, while the limit of detection for *P. falciparum* assay fluctuated from 0.3 to 8.0 parasites/µL (Supplementary Table 1). The reproducibility and repeatability of qPCR assays were high for both assays (Supplementary Figure 1A-B).

Supplementary Table 1. Sample characteristics and limit of detection for Pvr47 and Pfr364 assays in qPCR.

**Assay**	***Plasmodium* species**	**Sample**	**Parasitemia [a] (parasites/µL)**	**qPCR end-Point [b] (parasites/µL)**	**Mean Cq ± SD**
Pvr47	*P. vivax*	1	1944	0.9	36.1 ± 0.83
		2	9144	0.9	35.2 ± 1.59
		3	2448	0.9	38.1 ± 0.61
Pfr364	*P. falciparum*	4	1152	0.3	37.2 ± 0.56
		5	5112	8.0	36.1 ± 1.13
		6	5040	8.0	35.2 ± 0.77

Standard deviation, SD

[a] Parasitemia by LM.

[b] The lowest level of parasite density amplified by qPCR in 100% of five replicates. Genomic DNA were 3-fold serially diluted ranging from 220 to 0.3 parasites/µL.

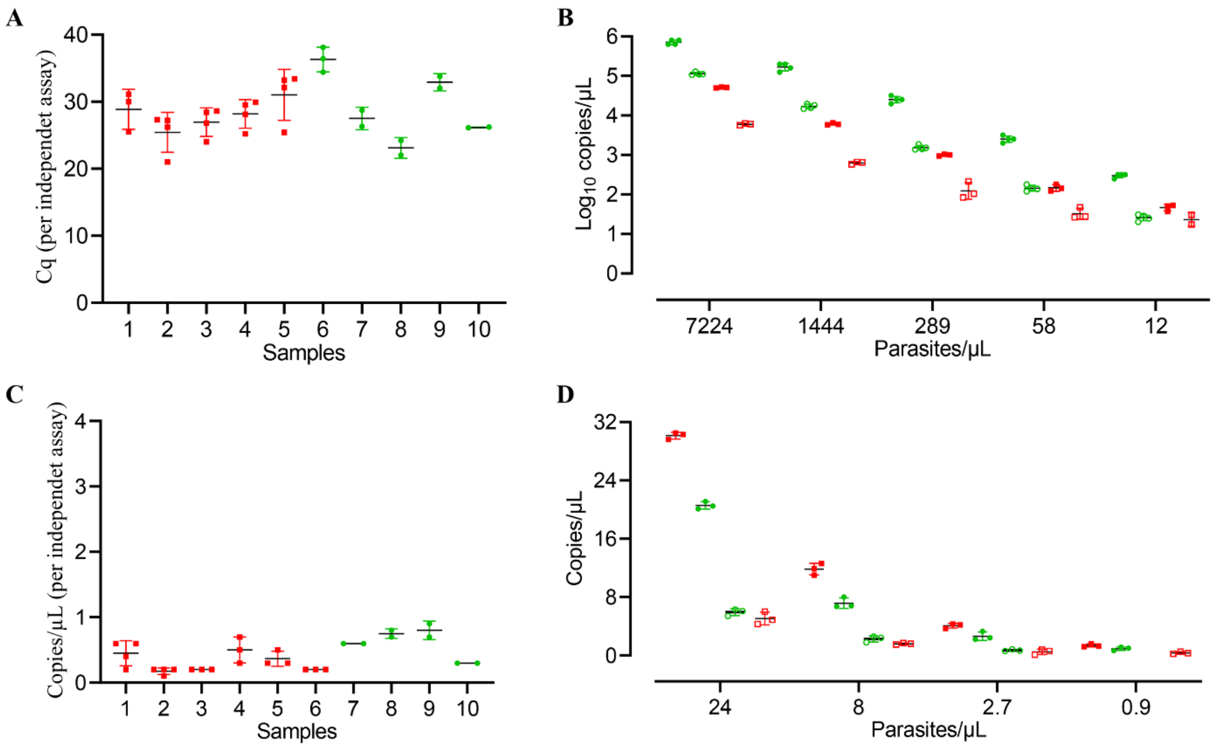

Supplementary Figure 1. Reproducibility and repeatability of qPCR and ddPCR. Cq values and concentration in copies/µL from independent assays for 10 samples were used to represent the reproducibility of qPCR and ddPCR **(A-C)**, respectively. The repeatability was represented by quantification of four DNA samples in triplicates within the same experiment of qPCR **(B)** and ddPCR **(D)**. The mean of each data set is shown as a solid line, error bars represent the standard deviation. *P. vivax*, in green and *P. falciparum*, in red

Agreement between parasite density estimates

To obtain the parasite density by qPCR as estimated by microscopy (parasite/µL), we initially determined the amount of copy numbers of plasmid DNA that is equivalent to parasite count in blood. Thus, the Cq values obtained from plasmid DNA curves were correlated with the Cq values obtained from clinical sample curves with known parasitemia by LM. The linear regression analysis indicated that 0.5 copies of plasmid DNA corresponds to 1 parasite/µL for *P. vivax* (Log_{10} parasite/µL = 0.744 Log_{10} Pvr47 copies – 0.327) (Suplementary Figure 2A). For *P. falciparum*, 1.7 copies of plasmid DNA is equivalent to 1 parasite/µL (Log_{10} parasite/µL = 0.758 Log_{10} Pfr364 copies + 0.227) (Suplementary Figure 2B).

For these samples, the geometric mean of parasite density detected by LM was 2137 parasites/µL (95% CI, 1328-3437 parasites/µL) for *P. vivax* and 1326 parasites/µL (95% CI, 771-2280 parasites/µL) for *P. falciparum*. There was moderate correlation between estimated parasite density by qPCR in blood and LM (r = 0.487, p = 0.0008 for *P. vivax*; r = 0.412, p = 0.006 by Spearman test for *P. falciparum*). Parasite density estimates by LM were approximately 5 to 10 times higher than parasitemia estimated by qPCR. The geometric mean parasite density estimated by qPCR was 222 parasites/µL (95% CI, 119-412 parasites/µL) and 256 parasites/µL (95% CI, 145-452 parasites/µL), respectively, for *P. vivax* and *P. falciparum*.

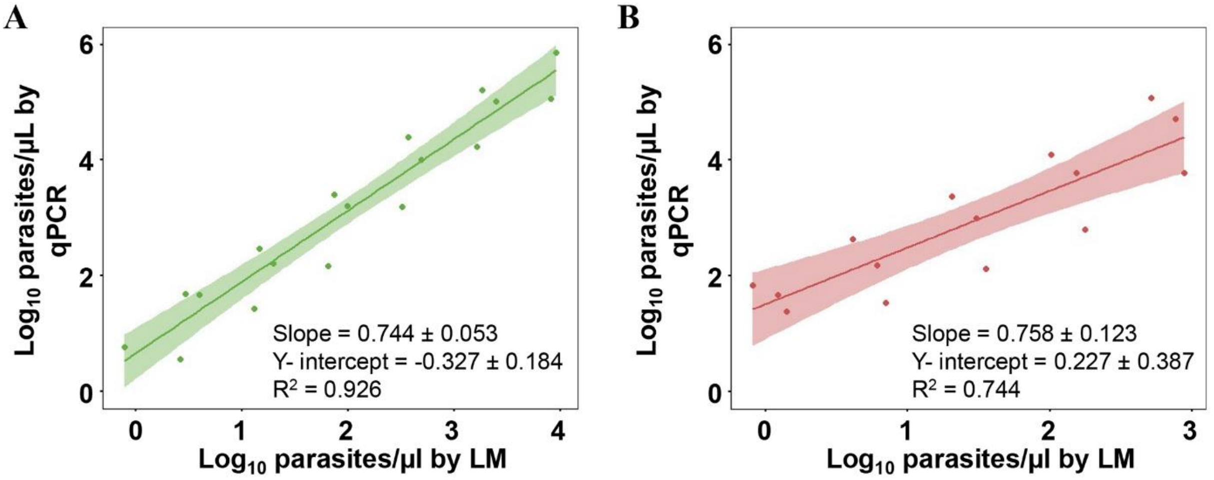

Supplementary Figure 2. Parasite density estimates by quantitative real-time PCR (qPCR). Linear regression analysis between parasitemia levels estimated by LM and qPCR (Log_{10} parasites/µL) for *P. vivax* **(A)** and *P. falciparum* samples **(B).** The shaded area represents the 95% confidence interval for the regression line.

Supplementary Tables

Supplementary Table 2. Results of 146 paired-samples of blood and saliva analyzed by qPCR.

	Saliva, n (%)				
**Blood**	***P. vivax***	***P. falciparum***	**Mixed** [a]	**Negative**	**Total**
P. vivax	48 (70.6)	2 (2.9)	0	18 (26.5)	68 (46.6)
P. falciparum	1 (2.5)	33 (82.5)	1 (2.5)	5 (12.5)	40 (27.4)
**Mixed**	5 (20.8)	5 (20.8)	6 (25.0)	8 (33.3)	24 (16.4)
**Negative**	2 (14.3)	1 (7.1)	5 (35.7)	6 (42.9)	14 (9.6)
**Total**	56 (38.4)	41 (28.1)	12 (8.2)	37 (25.3)	146 (100.0)

[a] Mixed-species infection (*P. vivax*/*P. falciparum*)

Supplementary Table 3. Assessment of sensitivity and specificity of diagnostic assays for 146 paired-samples in qPCR.

Protocol	True Positive [a]	False Positive [b]	False Negative	True Negative	Sensitivity, % (95% CI)	Especificity, % (95% CI)
**qPCR Blood**	109	23	8	6	93 (88-98)	21 (6-36)
**qPCR Saliva**	104	5	31	6	77 (70-84)	55 (26-84)
**qPCR Swab**	66	0	74	6	47 (39-55)	100 (100-100)
**LM**	120	1	20	5	86 (80-92)	83 (53-100)

[a] The reference standard (true positive) for each protocol was defined by combining the detections by any qPCR, excluding the protocol under evaluation.

[b] Infections not detected by any other molecular protocol.

Supplementary Table 4. Comparison of saliva samples storaged by different period of time and analyzed by qPCR.

Storage Time, N	Mean Cq (SD)	Interquartile range	Geometric mean Parasitemia (CI 95%)	*P* value [a]
<1 year (108)	34.4 (1.7)	32.6 – 35.0	1405 (947.2 – 2085)	0.1786
>1 year (71)	34.3 (0.8)	33.6 – 35.0	2498 (1776 – 3512)	

N, sample size

[a] Comparison of parasitemia carried out by Mann-Whitney test.

Supplementary Table 5. Sample characteristics and limit of detection for Pvr47 and Pfr364 assays in ddPCR.

**Assay**	**Species**	**Sample**	**Parasitemia [a] (parasites/μL)**	**ddPCR end-Point [b] (parasites/μL)**	**Parasite density [c] (copies/μL) ± SD**	**Parasite density [c] (copies/μL of blood) ± SD**
Pvr47	*P. vivax*	1	2448	0.9	0.9 ± 0.2	1.7 ± 0.4
		2	7272	2.7	0.7 ± 0.1	1.3 ± 0.2
Pfr364	*P. falciparum*	3	1152	0.1	0.2 ± 0.1	0.4 ± 0.1
		4	5112	0.9	0.4 ± 0.2	0.6 ± 0.3

[a] Parasitemia by LM.

[b] The lowest level of parasite density amplified by qPCR in 100% of three replicates. Genomic DNA were 3-fold serially diluted ranging from 24 to 0.1 parasites/μL and analyzed in triplicate.

[c] Parasite density estimated by ddPCR (mean ± standard deviation, SD).

Supplementary Table 6. Results of 86 paired-samples of blood and saliva analyzed by ddPCR.

	Saliva, n (%)				
**Blood**	***P. vivax***	***P. falciparum***	**Mixed** [a]	**Negative**	**Total**
P. vivax	17 (56.7)	3 (10.0)	1 (3.3)	9 (30.0)	30 (34.9)
P. falciparum	0	22 (75.9)	4 (13.8)	3 (10.3)	29 (33.7)
**Mixed**	4 (16.0)	5 (20.0)	5 (20.0)	11 (44.0)	25 (29.1)
**Negative**	0	1 (50.0)	0	1 (50.0)	2 (2.3)
**Total**	21 (24.4)	31 (36.0)	10 (11.6)	24 (27.9)	86 (100.0)

[a] Mixed-species infection (*P. vivax*/*P. falciparum*)

Supplementary Table 7. Assessment of sensitivity and specificity of diagnostic assays for 86 paired-samples in ddPCR.

Protocol	True Positive [a]	False Positive [b]	False Negative	True Negative	Sensitivity, % (95% CI)	Especificity, % (95% CI)
**ddPCR Blood**	73	11	1	1	99 (97-100)	8 (0-23)
**ddPCR Saliva**	62	0	23	1	73 (64-82)	100 (100-100)
**ddPCR Swab**	50	0	35	1	59 (49-69)	100 (100-100)
**LM**	76	1	9	0	89 (82-96)	0 (0-0)

[a] The reference standard (true positive) for each protocol was defined by combining the detections by any ddPCR, excluding the protocol under evaluation.

[b] Infections not detected by any other molecular protocol.

Supplementary Table 8. Comparison of saliva samples storaged by different period of time and analyzed by ddPCR.

Storage Time, N	Median Copies/µL (SD)	*P* value [a]	Interquartile range	Geometric mean Parasitemia (CI 95%)	*P* value [b]
**<1 year (77)**	4.5 (5.7)	0.068	1.1 – 13.0	1708 (1136 – 2567)	0.726
**>1 year (52)**	1.3 (1.8)		0.2 – 3.6	1966 (1326 – 2915)	

N, sample size

[a] Comparison of copies/µL performed by Mann-Whitney test.

[b] Comparison of parasitemia performed by Mann-Whitney test.
